# Distant organ metastasis patterns and prognosis of neuroendocrine cervical carcinoma: a population-based retrospective study

**DOI:** 10.3389/fendo.2022.924414

**Published:** 2022-08-16

**Authors:** Qing Li, Jie Yu, Hanjie Yi, Qiongyu Lan

**Affiliations:** ^1^ Department of Oncology, The Second Affiliated Hospital of Nanchang University, Nanchang, China; ^2^ Jiangxi Key Laboratory of Clinical and Translational Cancer Research, Nanchang, China; ^3^ Department of Oncology, Dongguan Tungwah Hospital, Dongguan, China

**Keywords:** neuroendocrine cervical carcinoma, SEER database, metastasis, prognosis, survival

## Abstract

**Background:**

Neuroendocrine carcinoma of the cervix (NECC) is a rare pathological form of cervical cancer. The prognosis of NECC with distant organ metastases is unclear. In our study, the patterns and prognosis of distant organ metastasis of NECC were investigated.

**Methods:**

Data were obtained from the surveillance epidemiology and end results (SEER) database from 2000 to 2018. Cox regression, Kaplan–Meier and log-rank analyses were conducted.

**Results:**

NECC was prone to single and multi-site metastases. The median overall survival (OS) was greatly decreased in patients with distant metastasis (*P* < 0.0001). Other characteristics such as age ≥60 years, poorer grade, higher T stage, those without surgery, no radiotherapy, and no chemotherapy were predictors of poor prognosis.

**Conclusions:**

Metastasis is an independent prognostic factor for patients with NECC. Surgery, radiotherapy, and chemotherapy give an overall survival advantage for patients with distant organ metastases.

## Background

Neuroendocrine carcinoma of the cervix (NECC) is a rare pathological type of cervical cancer, accounting for 1.4% of all cervical malignancies, and it has a poor prognosis (1). The fifth edition of the WHO 2020 divides neuroendocrine tumors are into highly differentiated neuroendocrine tumors and poorly differentiated neuroendocrine carcinoma (NEC). Among them, poorly differentiated NEC include small cell NEC and large cell NEC, with small cell NEC being the most common, accounting for about 80%, followed by large cell NEC (12%) and other histological types such as undifferentiated neuroendocrine neoplasms (8%) (1, 2).

The biology of NECC differs from that of squamous or adenocarcinoma of the cervix in that it exhibits a very aggressive biological behavior with a strong propensity for lymphatic and hematogenous spread. Local and distant recurrence is more common in NECC than in other pathological types of cervical cancer (3). Small cell NEC of the cervix is usually diagnosed at an advanced stage (4, 5), usually with metastasis to extra-pelvic organs such as the liver and lung as the first diagnosis (6). Its median overall survival (OS) is always less than 2 years (7, 8). At all stages of the disease, women with endocrine tumors have a worse survival rate compared to squamous cell carcinomas (5).

Due to the rarity of the disease, the optimal treatment for NECC remains uncertain. Current clinical experience mainly refers to the multimodal treatment of small cell lung cancer. The most common primary treatment modality for NECC is radical surgery combined with chemotherapy. Cohen et al. shown that adjuvant chemotherapy or chemoradiotherapy improved survival in patients with all stage of small cell NEC (7). The role of radiotherapy in NECC remains controversial, and Dong’s study showed that the combination of radiotherapy and surgery has a significant survival advantage in metastatic NECC (9). Due to the low incidence of NECC, fewer studies have been conducted on metastatic NECC, especially on distant organ metastases. In this paper, we focus on NECC patients with distant organ metastases from the surveillance epidemiology and end results (SEER) database to study the prognosis of different distant organ metastatic sites, and then to study the effect of surgery, radiotherapy, and chemotherapy on metastatic NECC.

## Methods

### Study cohorts

The data for this retrospective cohort study were obtained from the SEER database [Incidence-SEER 18 Regs Research Data, Nov 2020 Sub (2000–2018 varying)]. The SEER database collects information on cancer from 18 United states registries, representing close to 28% of the US population, and provides an oversized quantity of elaborated analysis data (10). Patients’ chemotherapy and radiotherapy records also are licensed to be used by the SEER workshop. The SEER*Stat software system (version 8.3.9, National Cancer Institute, Washington, USA) was used to access the data from the SEER database.

### Data collection

**Figure 1:**
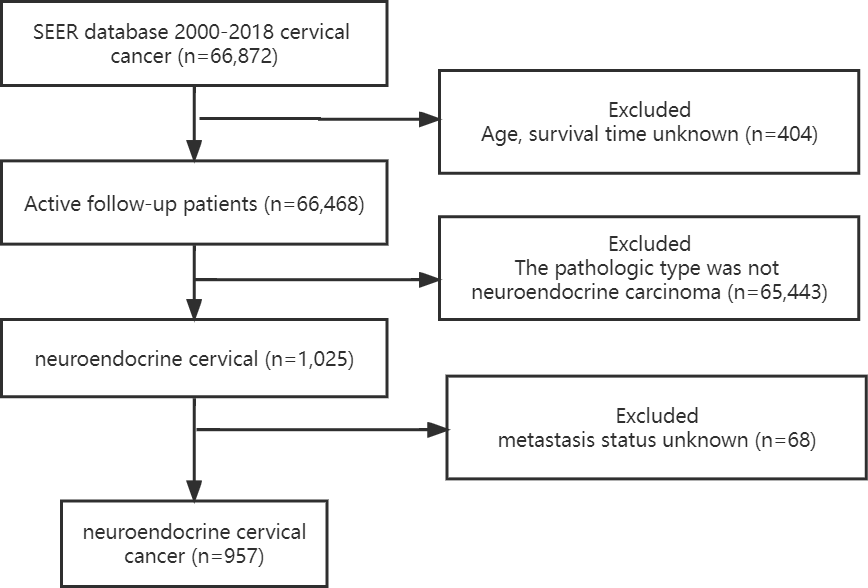
Flowchart of the neuroendocrine cervical cancer patient’s selection.

The NECC study data and relevant clinical information were obtained from the SEER project (1). Patients with first malignant primary site cervical cancer (Site record International Classification of Diseases for Oncology-3 (ICD-O-3)/WHO 2008: Cervix Uteri) diagnosed between 2000 and 2018 were identified from the SEER database (2). International Classification of Diseases for Oncology (ICD-O-03) histology codes of 8002, 8012-8014, 8031, 8041, 8043-8045, 8154, 8158, 8240-8246, 8249 and 8574 (3). Patients with unknown age, race, follow-up time, and metastatic status were excluded from participation. The flow chart for patient choice is shown in **Figure 1**.

Demographic characteristics were collected: age at diagnosis, race, marital status, AJCC stage, grade, tumor size, cancer metastasis, surgery of primary site, radiotherapy, chemotherapy, and survival status. These were designated as risk and/or prognosis factors for more analysis of NECC patients with metastatic NECC. The primary endpoint was OS.

### Statistical analysis

Descriptive analyses were performed on all participants. Categorical variables were expressed as proportions (%). Variables were compared using Pearson’s chi-square tests. Univariable and multivariable Cox regression analyses were used to assess the independent association between metastasis status and OS. Survival curves were plotted by Kaplan–Meier and log-rank analyses.

All the analyses were performed with the statistical software packages R (http://www.R-project.org, The R Foundation ) and Free Statistics software versions 1.5. A two-tailed test was performed and p < 0.05was considered statistically significant.

## Results

### Demographic and clinical characteristics for neuroendocrine cervical carcinoma

A total of 66,468 patients were diagnosed with cervical cancer between 2000 and 2018 according to the inclusion criteria, excluding patients with unknown age and survival time. A total of 1,025 neuroendocrine cervical cancer was collected by excluding patients with non-neuroendocrine type of pathology. After excluding patients with unknown distant metastasis status, a total of 957 cases remained. Detailed characteristics of the cohort of patients with neuroendocrine cervical cancer with and without distant metastases are listed in **Table 1**. The proportion of distant metastases was higher in middle-aged (41-60 years) and older (≥61 years) women than in young women (*P*<0.001). Other characteristics were different in patients with or without distant metastasis such as T, N, AJCC stage, tumor size, and metastatic organs including bone, brain, liver, and lung (*P*<0.001). A higher percentage of patients without distant metastases underwent primary site surgery (59.8% vs 20.7%, *P*<0.001), primary site radiation therapy (66.4% vs 44.9%, *P*<0.001), and chemotherapy (80.7% vs 74.5%, *P*<0.05).

**Table 1:** Demographic and clinical characteristics for neuroendocrine cervical carcinoma patients diagnosed with and without distant metastasis (2000–2018).

Subject characteristics	Total n (%) n = 957	M0 n (%) n = 565	M1 n (%) n = 392	*P* value
**Age(years)**				<0.001
≤40	304 (31.8)	226 (40)	78 (19.9)	
41-60	400 (41.8)	220 (38.9)	180 (45.9)	
≥61	253 (26.4)	119 (21.1)	134 (34.2)	
**Years of diagnosis (years)**				0.25
2000-2004	226 (23.6)	139 (24.6)	87 (22.2)	
2005-2009	214 (22.4)	135 (23.9)	79 (20.2)	
2010-2014	249 (26.0)	144 (25.5)	105 (26.8)	
2015-2018	268 (28.0)	147 (26)	121 (30.9)	
**Race**				0.317
black	140 (14.6)	84 (14.9)	56 (14.3)	
white	689 (72.0)	398 (70.4)	291 (74.2)	
other	128 (13.4)	83 (14.7)	45 (11.5)	
**Marital status**				0.061
married	416 (43.5)	251 (44.4)	165 (42.1)	
single	507 (53.0)	288 (51)	219 (55.9)	
unknown	34 (3.6)	26 (4.6)	8 (2)	
**Grade**				0.387
I	3 (0.3)	2 (0.4)	1 (0.3)	
II	17 (1.8)	12 (2.1)	5 (1.3)	
III	428 (44.7)	261 (46.2)	167 (42.6)	
IV	199 (20.8)	120 (21.2)	79 (20.2)	
unknown	310 (32.4)	170 (30.1)	140 (35.7)	
**T stage**				<0.001
T1	369 (38.6)	316 (55.9)	53 (13.5)	
T2	202 (21.1)	137 (24.2)	65 (16.6)	
T3	203 (21.2)	89 (15.8)	114 (29.1)	
T4	54 (5.6)	19 (3.4)	35 (8.9)	
Tx	129 (13.5)	4 (0.7)	125 (31.9)	
**N stage**				<0.001
N0	433 (45.2)	356 (63)	77 (19.6)	
N1	398 (41.6)	189 (33.5)	209 (53.3)	
Nx	126 (13.2)	20 (3.5)	106 (27)	
**AJCC Stage**				<0.001
I	252 (26.3)	252 (44.6)	0 (0)	
II	80 (8.4)	80 (14.2)	0 (0)	
III	213 (22.3)	213 (37.7)	0 (0)	
IV	409 (42.7)	19 (3.4)	390 (99.5)	
unknown	3 (0.3)	1 (0.2)	2 (0.5)	
**Tumor Size**				<0.001
<4cm	180 (18.8)	147 (26)	33 (8.4)	
≥4cm	363 (37.9)	207 (36.6)	156 (39.8)	
unknown	414 (43.3)	211 (37.3)	203 (51.8)	
**Bone metastasis**				<0.001
no	449 (46.9)	291 (51.5)	158 (40.3)	
yes	64 (6.7)	0 (0)	64 (16.3)	
unknown	444 (46.4)	274 (48.5)	170 (43.4)	
**Brain metastasis**				<0.001
no	499 (52.1)	291 (51.5)	208 (53.1)	
yes	14 (1.5)	0 (0)	14 (3.6)	
unknown	444 (46.4)	274 (48.5)	170 (43.4)	
**Liver metastasis**				<0.001
no	436 (45.6)	291 (51.5)	145 (37)	
yes	75 (7.8)	0 (0)	75 (19.1)	
unknown	446 (46.6)	274 (48.5)	172 (43.9)	
**Lung metastasis**				<0.001
no	430 (44.9)	291 (51.5)	139 (35.5)	
yes	81 (8.5)	0 (0)	81 (20.7)	
unknown	446 (46.6)	274 (48.5)	172 (43.9)	
**Primary surgery**				<0.001
yes	419 (43.8)	338 (59.8)	81 (20.7)	
no	538 (56.2)	227 (40.2)	311 (79.3)	
**Radiotherapy**				<0.001
yes	551 (57.6)	375 (66.4)	176 (44.9)	
no	406 (42.4)	190 (33.6)	216 (55.1)	
**Chemotherapy**				0.027
yes	748 (78.2)	456 (80.7)	292 (74.5)	
no	209 (21.8)	109 (19.3)	100 (25.5)	

The distant metastasis (M1) and no distant metastasis (M0) arms of the study were stratified based on the age of diagnosis, race, marital status, differentiation grade, AJCC stage, T stage, N stage, tumor size, treatments received (surgery, radiotherapy, chemotherapy). The two groups were subject to Pearson’s Chi-square statistical analysis.

### Frequency of organ metastasis

The distribution of distant metastatic sites is shown in **Table 2**. 142 patients were able to extract data describing specific metastatic sites from the SEER database since 2010. Single-site metastases accounted for 55.6% of the cases, with liver metastases being the most common site of metastasis (20.4%), followed by lung (19.7%) and bone (13.4%) metastases. A total of 44.4% of patients had multi-organ metastases. Metastases to three organ, lung, liver, and bone, were more common than other multi-organ metastases (11.3%).

**Table 2:** Frequencies of different metastasis sites and combination metastasis (n=142).

Metastatic site		Number	Percentage (%)
One site	Bone	19	13.4
	Brain	3	2.1
	Liver	29	20.4
	Lung	28	19.7
	Total	79	55.6
Two sites	Lung and liver	14	9.9
	Lung and bone	13	9.2
	Lung and brain	1	0.7
	Liver and bone	8	5.6
	Liver and brain	1	0.7
	bone and brain	1	0.7
	Total	38	26.8
Three sites	Lung and liver and bone	16	11.3
	Lung and liver and brain	2	1.4
	Lung and bone and brain	3	2.1
	Liver and bone and brain	2	1.4
	Total	23	16.2
Four sites	all	2	1.4

### Cox proportional hazards regression analysis

Univariate and multivariate Cox regression was used to distinguish potential prognostic factors for OS (**Table 3**) and CSS (**Supplementary Table S1**) in patients with NECC with or without distant metastases. In the univariate analysis, we identified patients in the age group 41-60 years and above, white race, single status, higher T, N, M and AJCC stages, tumor size≥4cm, number of organ metastasis. In the group without distant organ metastases, 59.8% of patients received surgery, 66.4% received radiotherapy, and 80.7% received chemotherapy. However, in the distant metastasis group, only 20.7% of patients underwent surgery, 44.9% received radiotherapy, and 74.5% received chemotherapy. In the multivariable COX regression analysis, distant metastasis remained a risk factor for poor prognosis (HR: 1.85, 95%CI: 1.01-3.38, *P*<0.05). Other characteristics such as age older than 60 years old (HR: 1.66, 95%CI:1.32-2.07, *P*<0.001), worse grade, higher T stage, those without surgery (HR: 1.9, 95%CI: 1.54-2.35, *P*<0.001), no radiotherapy (HR: 1.29, 95%CI: 1.08-1.55, *P*=0.006), and no chemotherapy (HR: 2.7, 95%CI: 2.21-3.3, *P*<0.001) were predictors of poor prognosis. A similar survival trend was also observed for CSS (**Supplementary Table S1**).

**Table 3:** Univariable and multivariable Cox regression analysis of overall survival in neuroendocrine cervical cancer patients with metastasis in SEER database (2000–2018).

Subject characteristics	Univariable		Multivariable	
	HR (95% CI)	*P-*value	HR (95% CI)	*P-*value
**M stage**				
M0	Ref	1.0	Ref	1.0
M1	3.58 (3.06,4.2)	<0.001**	1.85 (1.01~3.38)	0.045*
**Age(years)**				
≤40	Ref	1.0	Ref	1.0
41-60	1.67 (1.38,2.02)	<0.001**	1.2 (0.98~1.47)	0.084
≥61	2.95 (2.4,3.62)	<0.001**	1.66 (1.32~2.07)	<0.001**
**Race**				
black	Ref	1.0	Ref	1.0
white	0.72 (0.58,0.89)	0.002	0.96 (0.77~1.2)	0.717
other	0.66 (0.5,0.88)	0.004*	0.86 (0.64~1.16)	0.325
**Marital status**				
married	Ref	1.0	Ref	1.0
single	1.29 (1.1,1.51)	0.001**	1.03 (0.87~1.21)	0.766
unknown	0.83 (0.52,1.32)	0.426	0.89 (0.55~1.43)	0.626
**Grade**				
I	Ref	1.0	Ref	1.0
II	0.25 (0.06,1.14)	0.074	0.22 (0.05~1.02)	0.054
III	0.33 (0.08,1.32)	0.117	0.21 (0.05~0.85)	0.029*
IV	0.33 (0.08,1.35)	0.124	0.21 (0.05~0.86)	0.03*
unknown	0.36 (0.09,1.46)	0.153	0.18 (0.04~0.76)	0.019*
**T stage**				
T1	Ref	1.0	Ref	1.0
T2	2.11 (1.69,2.63)	<0.001**	1.49 (1.1~2.02)	0.011**
T3	3.63 (2.93,4.49)	<0.001**	1.78 (1.34~2.38)	<0.001**
T4	5.95 (4.32,8.21)	<0.001**	2.25 (1.45~3.5)	<0.001**
Tx	5.24 (4.12,6.67)	<0.001**	1.67 (1.2~2.34)	0.003*
**N stage**				
N0	Ref	1.0	Ref	1.0
N1	2.1 (1.77,2.49)	<0.001**	1.15 (0.91~1.46)	0.243
Nx	3.77 (3,4.74)	<0.001**	1.2 (0.9~1.61)	0.222
**AJCC Stage**				
I	Ref	1.0	Ref	1.0
II	1.84 (1.29,2.61)	<0.001**	1.11 (0.68~1.8)	0.674
III	2.52 (1.96,3.26)	<0.001**	1.64 (1.11~2.41)	0.013*
IV	6.15 (4.89,7.73)	<0.001**	1.51 (0.76~3.01)	0.242
unknown	5.99 (1.47,24.39)	0.012*	1.83 (0.38~8.8)	0.45
**Tumor Size**				
<4cm	Ref	1.0	Ref	1.0
≥4cm	1.93 (1.52,2.47)	<0.001**	0.97 (0.74~1.26)	0.806
unknown	2.07 (1.63,2.63)	<0.001**	0.89 (0.68~1.17)	0.416
**Number of metastasis**				
No metastasis	Ref	1.0	Ref	1.0
1 site metastasis	3.22 (2.42,4.28)	<0.001**	1.14 (0.83~1.57)	0.402
≥1 sites metastasis	3.82 (2.82,5.18)	<0.001**	1.62 (1.15~2.27)	0.005*
unknown	1.45 (1.21,1.73)	<0.001**	1.19 (0.97~1.45)	0.095
**Primary site surgery**				
yes	Ref	1.0	Ref	1.0
no	3.04 (2.57,3.59)	<0.001**	1.9 (1.54~2.35)	<0.001**
**Radiotherapy**				
yes	Ref	1.0	Ref	1.0
no	1.38 (1.18,1.61)	<0.001**	1.29 (1.08~1.55)	0.006*
**Chemotherapy**				
yes	Ref	1.0	Ref	1.0
no	2.07 (1.74,2.47)	<0.001**	2.7 (2.21~3.3)	<0.001**

HR, Hazard Ratio; CI, Confidence Interval; Ref, reference.

*P<0.05; **P<0.001.

Stratified analyses to evaluate the relationships between distant metastases and mortality in the different subgroups. Distant metastasis NECC have poor prognosis in each subgroup (**Supplementary Table S2**).

### Effects of treatment on NECC patients with or without metastasis

**Figure 2:**
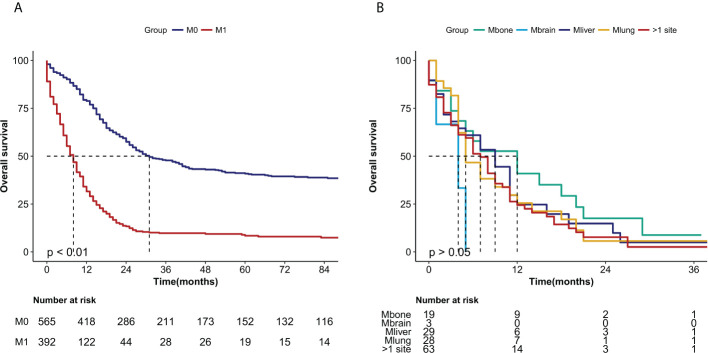
Kaplan-Meier curves of the overall survival in NECC cancer stratified by **(A)** metastasis (M0: no metastasis, M1 metastasis) (*P* < 0.0001) and **(B)** different sites of metastasis (M bone, M brain, M liver, M lung, M >1 site) (*P* > 0.05).

OS curves were showed in **Figure 2**. The median overall survival months were considerably lower in patients with distant metastases (30 months vs 7 months, *P*<0.0001) (**Figure 2A**). However, there was no statistically significant difference in median OS for metastases from different organs such as bone, brain, liver, lung and metastases from more than one site (*P*>0.05) (**Figure 2B**).

**Figure 3:**
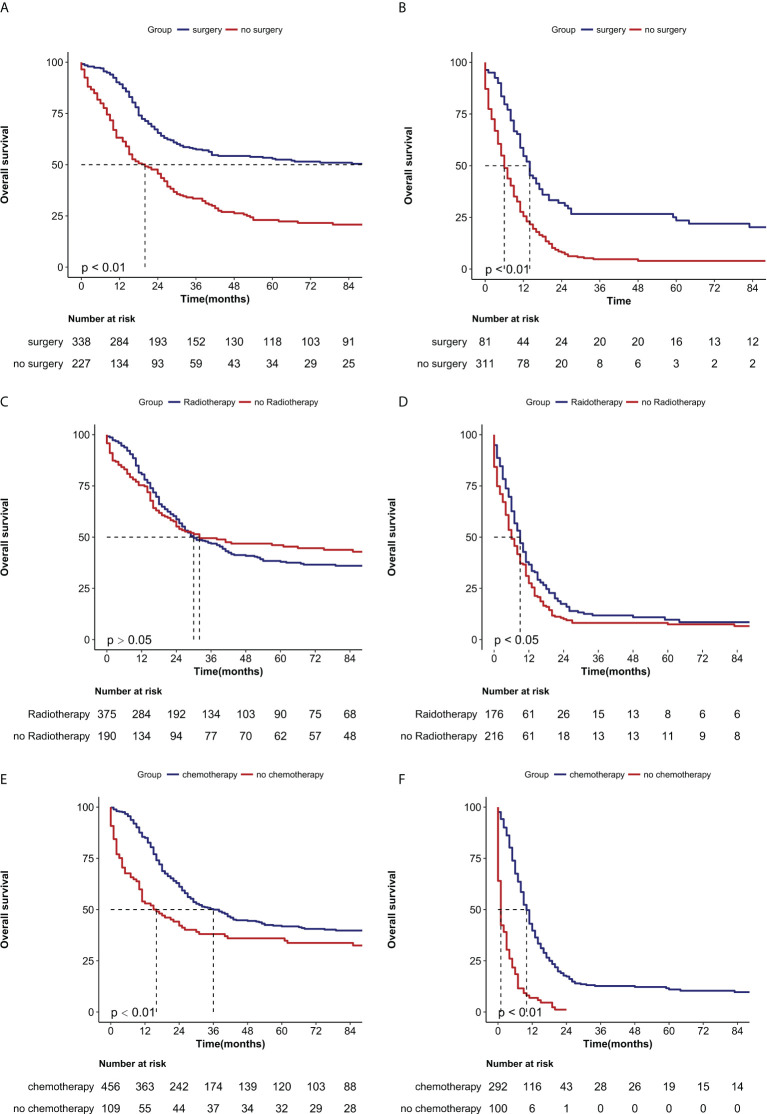
Kaplan-Meier curves of the overall survival in NECC with no metastasis or metastasis when stratified by treatment. **(A)** Surgery in no metastasis patients (*P *< 0.0001); **(B)** Surgery in metastasis patients (*P* < 0.0001) **(C)** Radiotherapy in no metastasis patients (*P* > 0.05), **(D)** Radiotherapy in metastasis patients (*P* < 0.05), **(E)** Chemotherapy in no metastasis patients (*P* < 0.001) **(F)** Chemotherapy in metastasis patients (*P* < 0.0001).

To investigate the therapeutic effect of treatment on patients with NECC, we compared the survival effects of surgery, radiotherapy, and chemotherapy on patients with and without metastases. Patients who were treated with surgery had median survival months of 112 months compared to 19 months for patients who were not treated with surgery in the M0 group (*P*<0.0001) (**Figure 3A**). Even among people with distant metastases, the median survival was 13 months in the surgery group and 5 months in the no-surgery group, respectively (*P*<0.0001) (**Figure 3B**). In the group of patients without metastases, there was no statistical difference in OS between those who received radiotherapy and those who did not (*P*>0.05) (**Figure 3C**). However, In the distant metastasis group, there was a statistical difference in OS between the radiotherapy and no radiotherapy groups (median of 13 months vs 5 months, *P*<0.05) (**Figure 3D**). when patients received chemotherapy, there was a significant difference in the median OS in both the no metastasis (37 months vs 15 months, P=0.0005) **Figure 3E**) and the metastasis groups (9 months vs 1 month, *P*<0.0001) compared with the no-chemotherapy group (**Figure 3F**).

## Discussion

Compared to adenocarcinoma and squamous cell carcinoma, patients with small cell NEC have the worst prognosis in both the early and advanced stages of cancer (11). Due to its highly aggressive nature, lymph node metastases and distant metastases often occur at an early stage. In this SEER study, 42.7% of patients were diagnosed with stage IV. However, due to the rare incidence of NECC, there are very few studies on distant organ metastatic NECC. Our study showed that distant organ metastasis was more likely to occur in patients older than 40 years, with T3 or T4 stage, tumor size ≥4cm, and with lymph node metastasis N1 at diagnosis.

The metastasis pattern of NECC differs from that of other pathological types of cervical cancer. Previous studies have found that lung metastasis and bone metastasis are the most common organs of metastasis in squamous cell carcinoma of the uterine cervix (12). Bone, brain, liver, and bone marrow are the most common distant metastatic sites for small cell NEC (4, 13). With our limited data from the SEER database after 2010, the most common single metastatic site for NECC was the liver (20.4%), followed by the lung (19.7%). What’s more, multisite metastases were very common in patients with NECC, with 44.4% of patients in our study presenting with metastases from more than one site. 26.8% of patients presented with metastases from two sites, and 17.6% of patients presented with metastases from three or more organs. The median OS for single bone metastases was longest but less than 12 months, compared with a median OS of 6 months for multiple metastases. The worst prognosis is for brain metastases alone, with a median OS of less than 5 months. However, due to the small sample size, we need further studies to confirm this conclusion.

For patients without distant organ metastases, primary site surgery and radiotherapy are mostly used, whereas chemotherapy is mostly used for patients with distant organ metastases. Cohen et al. summarized the results of 188 patients in which the use of adjuvant chemotherapy or chemoradiotherapy was associated with high survival rate in patients with early small cell NEC (7). External beam radiotherapy coupled with brachytherapy has been shown to improve median survival in locally advanced NECC (14). Lin et al. showed that radical surgery should be recommended for early-stage small cell NEC and a combination of radiotherapy and brachytherapy should be used for patients with advanced disease (15). However, Hou’s study showed that radical surgery versus primary RT did not affect the survival of high-grade NECC (16). Studies on the treatment of distant organ metastatic NECC are limited. In a study of patients with relapsed NECC, the use of the combination of topotecan, paclitaxel, and bevacizumab (TPB) improved progression-free survival whit a trend toward improved OS (17). In our study, of those NECC patients with distant metastases, 20.7% received primary site surgery, 44.9% received radiotherapy, and 74.5% received chemotherapy. Furthermore, primary site surgery, radiotherapy, and chemotherapy improved OS in patients with metastatic NECC.

Since this study is a retrospective study based on the SEER database, there are some limitations in our study. First, based on the SEER database, inherent selection bias is inevitable, and we could only try to control for confounding factors. Second, metastatic sites other than brain, bone, liver, and lung were not included, and the specific organ transfer sites were unknown before 2010. In addition, there is no standard chemotherapy regimen for NECC, Cisplatin/carboplatin and etoposide (EP) are by far the most used treatment regimens (1, 18). However, the lack of specific chemotherapy information in the SEER database, we were unable to evaluate specific chemotherapy regimen. Similarly, we lack information on specific sites and methods of radiotherapy as well as targeted therapies and immunotherapy for NECC. We believe that patients with metastatic NECC can benefit from systemic chemotherapy and aggressive local treatment such as surgery, and radiotherapy. Hence, more randomized trials for patients with distant organ NECC are needed.

## Conclusions

Our results showed that NECC is prone to a single-site and multi-site metastases, with the most common site of metastasis being the liver, followed by the lung. Median OS was less than 12 months in all metastatic patients, but metastases at different sites or multi-organ metastases did not affect OS. Metastases, age, T stage, surgery, radiotherapy, and chemotherapy were independent prognostic factors for patients with NECC. Surgery, radiotherapy, and chemotherapy all provide benefits to patients with distant organ metastases.

## Data availability statement

The raw data supporting the conclusions of this article will be made available by the authors, without undue reservation.

## Author contributions

QL designed this study, analyzed the data, and wrote the manuscript. HY and JY contributed to the analysis of the data QYL reviewed the manuscript. All authors contributed to the article and approved the submitted version.

## Funding

This Study was funded by grants from Jiangxi youth science fund project in China (grant number 20181BAB215029) and the National Natural Science Foundation of China (Grand No. 81960550).

## Conflict of interest

The authors declare that the research was conducted in the absence of any commercial or financial relationships that could be construed as a potential conflict of interest.

## Publisher’s note

All claims expressed in this article are solely those of the authors and do not necessarily represent those of their affiliated organizations, or those of the publisher, the editors and the reviewers. Any product that may be evaluated in this article, or claim that may be made by its manufacturer, is not guaranteed or endorsed by the publisher.
