# Supplementary material for: Distant organ metastasis patterns and prognosis of neuroendocrine cervical carcinoma: a population-based retrospective study
Source: Front Endocrinol (Lausanne). 2022 Aug 16;13:924414. doi: 10.3389/fendo.2022.924414 (PMC9424674; doi:10.3389/fendo.2022.924414)
Supplement: Supplementary file 1 [file Table_1.docx]

**Supplement table 1** Univariable and multivariable Cox regression analysis of cause-specific survival in neuroendocrine cervical cancer patients with metastasis in SEER database (2000–2018)

| **Subject characteristics** | **Univariable**  **HR (95% CI)** | | ***P-*value** | **Multivariable**  **HR (95% CI)** | ***P*-value** |
| --- | --- | --- | --- | --- | --- |
| **M stage** | |  |  |  |  |
| M0 | | Ref | 1.0 | Ref | 1.0 |
| M1 | | 3.62 (3.07~4.28) | < 0.001 | 1.91 (1.01~3.61) | 0.048 |
| **Age(years)** | |  |  |  |  |
| ≤40 | | Ref | 1.0 | Ref | 1.0 |
| 41-60 | | 1.58 (1.3~1.92) | < 0.001 | 1.1 (0.89~1.35) | 0.384 |
| ≥61 | | 2.61 (2.11~3.23) | < 0.001 | 1.42 (1.13~1.8) | 0.003 |
| **Race** | |  |  |  |  |
| black | | Ref | 1.0 | Ref | 1.0 |
| white | | 0.7 (0.57~0.87) | 0.001 | 0.91 (0.72~1.14) | 0.398 |
| other | | 0.61 (0.45~0.83) | 0.002 | 0.79 (0.57~1.08) | 0.133 |
| **Marital status** | |  |  |  |  |
| married | | Ref | 1.0 | Ref | 1.0 |
| single | | 1.29 (1.09~1.51) | 0.003 | 1.03 (0.86~1.23) | 0.755 |
| unknown | | 0.8 (0.49~1.3) | 0.367 | 0.85 (0.52~1.41) | 0.54 |
| **Grade** | |  |  |  |  |
| I | | Ref | 1.0 | Ref | 1.0 |
| II | | 0.48 (0.06~3.75) | 0.483 | 0.44 (0.06~3.58) | 0.446 |
| III | | 0.63 (0.09~4.53) | 0.65 | 0.43 (0.06~3.14) | 0.407 |
| IV | | 0.64 (0.09~4.61) | 0.66 | 0.43 (0.06~3.14) | 0.405 |
| unknown | | 0.68 (0.09~4.84) | 0.697 | 0.37 (0.05~2.69) | 0.325 |
| **T stage** | |  |  |  |  |
| T1 | | Ref | 1.0 | Ref | 1.0 |
| T2 | | 2.25 (1.79~2.83) | < 0.001 | 1.64 (1.19~2.25) | 0.002 |
| T3 | | 3.77 (3.01~4.72) | < 0.001 | 1.95 (1.45~2.64) | <0.001 |
| T4 | | 5.79 (4.11~8.15) | < 0.001 | 2.24 (1.4~3.59) | 0.001 |
| Tx | | 5.59 (4.35~7.19) | < 0.001 | 1.81 (1.27~2.57) | 0.001 |
| **N stage** | |  |  |  |  |
| N0 | | Ref | 1.0 | Ref | 1.0 |
| N1 | | 2.12 (1.77~2.54) | < 0.001 | 1.14 (0.89~1.47) | 0.291 |
| Nx | | 4.07 (3.22~5.15) | < 0.001 | 1.29 (0.95~1.74) | 0.103 |
| **AJCC Stage** | |  |  |  |  |
| I | | Ref | 1.0 | Ref | 1.0 |
| II | | 1.92 (1.33~2.78) | < 0.001 | 1.11 (0.67~1.83) | 0.697 |
| III | | 2.62 (2~3.42) | < 0.001 | 1.64 (1.09~2.47) | 0.017 |
| IV | | 6.35 (4.99~8.09) | < 0.001 | 1.49 (0.72~3.09) | 0.286 |
| unknown | | 6.72 (1.65~27.42) | 0.008 | 1.89 (0.39~9.26) | 0.43 |
| **Tumor Size** | |  |  |  |  |
| <4cm | | Ref | 1.0 | Ref | 1.0 |
| ≥4cm | | 2 (1.55~2.58) | < 0.001 | 1.01 (0.76~1.33) | 0.958 |
| unknown | | 2.17 (1.69~2.78) | < 0.001 | 0.93 (0.7~1.24) | 0.634 |
| **Number of metastasis** | |  |  |  |  |
| No metastasis | | Ref | 1.0 | Ref | 1.0 |
| 1 site metastasis | | 3.32 (2.47~4.46) | < 0.001 | 1.21 (0.87~1.68) | 0.257 |
| ≥1 sites metastasis | | 3.74 (2.72~5.16) | < 0.001 | 1.63 (1.14~2.32) | 0.007 |
| unknown | | 1.51 (1.26~1.82) | < 0.001 | 1.22 (0.99~1.51) | 0.061 |
| **Primary site surgery** | |  |  |  |  |
| yes | | Ref | 1.0 | Ref | 1.0 |
| no | | 2.88 (2.42~3.42) | < 0.001 | 1.72 (1.39~2.13) | <0.001 |
| **Radiotherapy** | |  |  |  |  |
| yes | | Ref | 1.0 | Ref | 1.0 |
| no | | 1.38 (1.17~1.62) | < 0.001 | 1.28 (1.06~1.55) | 0.011 |
| **Chemotherapy** | |  |  |  |  |
| yes | | Ref | 1.0 | Ref | 1.0 |
| no | | 2.06 (1.72~2.48) | < 0.001 | 2.68 (2.18~3.31) | <0.001 |

HR: Hazard Ratio; CI: Confidence Interval
