# Supplementary material for: Distant organ metastasis patterns and prognosis of neuroendocrine cervical carcinoma: a population-based retrospective study
Source: Front Endocrinol (Lausanne). 2022 Aug 16;13:924414. doi: 10.3389/fendo.2022.924414 (PMC9424674; doi:10.3389/fendo.2022.924414)
Supplement: Supplementary file 2 [file Table_2.docx]

**Supplement Table 2** Subgroup analyses of the association of overall survival in neuroendocrine cervical cancer patients with metastasis

| **Confounding factor category** | **HR (95% CI)** | | ***P for Trend*** | ***P for Interaction*** |
| --- | --- | --- | --- | --- |
|  | **M0** | **M1** |  |  |
| **Years of diagnosis (years)** |  |  |  | 0.006* |
| 2000-2009 | 1(Ref) | 2.9 (2.25~3.74) | <0.001** |  |
| 2010-2018 | 1(Ref) | 2.05 (1.46~2.88) | <0.001** |  |
| **Age(years)** |  |  |  | 0.001** |
| ≤40 | 1(Ref) | 4.32 (2.9~6.43) | <0.001** |  |
| 41-60 | 1(Ref) | 2.72 (2.02~3.67) | <0.001** |  |
| ≥61 | 1(Ref) | 1.65 (1.15~2.38) | 0.007* |  |
| **Primary surgery** |  |  |  | 0.520 |
| yes | 1(Ref) | 2.43 (1.71~3.45) | <0.001** |  |
| no | 1(Ref) | 2.34 (1.83~2.99) | <0.001** |  |
| **Radiotherapy** |  |  |  | 0.061 |
| yes | 1(Ref) | 2.73 (2.13~3.5) | <0.001** |  |
| no | 1(Ref) | 2.18 (1.52~3.13) | <0.001** |  |
| **Chemotherapy** |  |  |  | <0.001** |
| yes | 1(Ref) | 2.28 (1.82~2.86) | <0.001** |  |
| no | 1(Ref) | 3.4 (2.22~5.23) | <0.001** |  |

HR: Hazard Ratio; CI: Confidence Interval; Ref: reference.

**P*<0.05; ***P*<0.001
